# Phenotypic variation of *FXN* compound heterozygotes in a Friedreich ataxia cohort

**DOI:** 10.1002/acn3.52027

**Published:** 2024-02-23

**Authors:** Megan M. Shen, Christian Rummey, David R. Lynch

**Affiliations:** ^1^ Division of Neurology The Children's Hospital of Philadelphia Philadelphia Pennsylvania USA; ^2^ Perelman School of Medicine, University of Pennsylvania. Philadelphia Pennsylvania USA; ^3^ Clinical Data Science GmbH Basel Switzerland

## Abstract

**Objective:**

Most individuals with Friedreich ataxia (FRDA) have homozygous GAA triplet repeat expansions in the *FXN* gene, correlating with a typical phenotype of ataxia and cardiomyopathy. A minority are compound heterozygotes carrying a GAA expansion on one allele and a mutation on the other. The study aim was to examine phenotypic variation among compound heterozygotes.

**Methods:**

Data on *FXN* mutations were obtained from the Friedreich Ataxia Clinical Outcome Measures Study (FA‐COMS). We compared clinical features in a single‐site FA‐COMS cohort of 51 compound heterozygous and 358 homozygous patients, including quantitative measures of cardiac, neurologic, and visual disease progression.

**Results:**

Non‐GAA repeat mutations were associated with reduced cardiac disease, and patients with minimal/no function mutations otherwise had a typical FRDA phenotype but with significantly more severe progression. The partial function mutation group was characterized by relative sparing of bulbar and upper limb function, as well as particularly low cardiac involvement. Other clinical features in this group, including optic atrophy and diabetes mellitus, varied widely depending on the specific type of partial function mutation.

**Interpretation:**

These data support that the typical FRDA phenotype is driven by frataxin deficiency, especially severe in compound heterozygotes with minimal/no function mutations, whereas the heterogeneous presentations of those with partial function mutations may indicate other contributing factors to FRDA pathogenesis.

## Introduction

Friedreich ataxia (FRDA) is an autosomal recessive disorder that is clinically characterized by progressive ataxia, hypertrophic cardiomyopathy, scoliosis, and less commonly diabetes and optic atrophy.^1^, ^2^, ^3^ The vast majority (~96–98% by prior estimates) of individuals diagnosed with FRDA have homozygous GAA repeat expansions in intron 1 of the *FXN* gene, with the remainder having a repeat expansion on one allele with a mutation on the other allele.^1^, ^2^, ^3^, ^4^ More than 60 different mutations in frataxin have been described in compound heterozygotes.^5^ Many of these mutations result in truncation or minimal production of functional protein, which has been associated with a more severe disease course and thought to be driven by particularly low residual frataxin levels.^5^, ^6^, ^7^, ^8^ Other mutations with less destabilizing effects on frataxin, have more variable presentations that do not reliably fit the typical FRDA phenotype.^5^, ^6^, ^7^, ^8^, ^9^, ^10^, ^11^, ^12^

More recently, an individual born to consanguineous parents has been found to have homozygous R165C mutations in both *FXN* alleles, which challenged long‐standing theories that two *FXN* mutations would be embryonically lethal.^13^ His phenotype includes early vision loss, motor neuropathy with relatively mild ataxia, and absent cardiac disease; his clinical diagnosis was Charcot‐Marie‐Tooth (CMT) disease, reinviting previous speculations on phenotypic overlap between CMT and FRDA.^14^, ^15^

Frataxin has classically been understood to have a primary role in Fe‐S cluster synthesis and targets to the mitochondria, with frataxin deficiency resulting in impaired ATP production.^16^, ^17^ The most common partial function variant G130V, and I154F, impair frataxin processing (and likely other cellular events) and may cause a buildup of precursor or intermediate forms.^18^ Other functions of frataxin remain an active area of investigation as the clinical heterogeneity associated with the *FXN* gene is still not well understood. Some potential roles include those related to its interactions with other proteins such as GRP75, as well as with mitochondrial enzymes.^19^, ^20^, ^21^

Much of the literature on compound heterozygotes in FRDA has been reported as part of case reports or series; a few summative clinical studies have primarily focused on either descriptive data or binary measures (i.e., presence or absence of specific features).^5^, ^6^, ^9^ For example, compound heterozygotes have been described to have increased diabetes mellitus and decreased cardiomyopathy but with sparse detail.^5^ Here we present a comprehensive study of clinical features among a single‐site cohort of compound heterozygotes, with the characterization of quantitative metrics that include cardiac, neurologic, and visual domains. These findings have implications for not only the management of disease in these individuals but also potential insights into mechanisms of frataxin dysfunction underlying FRDA.^22^

## Methods

### Patient cohort

Data on compound heterozygotes were collected from the Friedreich Ataxia –Clinical Outcome Measures Study (FA‐COMS), to report on all known *FXN* mutations in this international multisite natural history study. For all other analyses, the sample cohort included all patients evaluated at the Children's Hospital of Philadelphia between February 2020 and April 2023. Nine additional compound heterozygote individuals seen before this time window were also included, age‐adjusted to reflect their most recent visit. The Institutional Review Board at the Children's Hospital of Philadelphia approved all protocols (2609, 7235, and 5852).

### Clinical measures

Baseline demographics and characteristics were recorded in FA‐COMS as previously described,^26^ including genetic testing information by commercial or research reports, sex, age of FRDA onset, modified Friedreich Ataxia Rating Scale (mFARS) components, medications, and patient‐reported disease features or symptoms (cardiomyopathy, diabetes, optic atrophy, scoliosis, arrhythmia, and hearing loss). Mutations were divided into minimal/no function or partial function groups based on previous modeling of frataxin and literature review of clinical reports^5^, ^6^, ^7^, ^8^, ^9^, ^18^, ^22^, ^23^, ^24^, ^25^ or conceptual predictions when empiric data were lacking. Mutations with unknown in vitro features and unclear predicted effects, including those in splice sites, were classified as partial function. Some mutations were not classified if not present in the single‐site cohort analyses. Whole blood and buccal cell samples were collected and analyzed as previously reported.^27^ Disease duration was defined as the difference between age and the age of FRDA onset, and disease burden for analyses was defined as the product of the disease duration and the GAA1 repeat expansion number (shorter repeat, if homozygous).

Chart review was performed to obtain the most recent troponin level, if available, as well as more detailed information on clinical and cardiac features in heterozygote patients. Troponin was considered elevated in low‐sensitivity assays at >0.03 ng/mL, and in high‐sensitivity assays at >20 ng/L for male and >15 ng/L for female sex, approximately based on 99th percentile ranges.^28^ The most recent available study was used for echocardiogram, ECG, and Holter monitor data by chart review, excluding those measured in the setting of acute illness. The biplane assessment of ejection fraction (EF) was used, and the E/e′ ratio was averaged between the medial/septal and lateral measurements, when available. Optical coherence tomography (OCT) data were collected as previously reported.^29^ Retinal nerve fiber layer (RFNL) thickness was reported as the average of the measurements from the left and right eyes. Neurological progression was assessed using the long‐term natural history data of the mFARS and the FARS E.

### Statistical analyses

Data analysis was performed using STATA version 18 (College Station, TX, USA) and R version 4.1.3. *χ* ^2^ tests were used for categorical data with one‐way ANOVA tests with Bonferroni correction were used for continuous data. Logistical regression was used for multivariate analysis, with statistical significance considered at *P* < 0.05 for all analyses. Random coefficient regression models were fitted using lmer4, as previously reported.^30^

## Results

### Clinical variation by FXN mutation type and subtypes

The FA‐COMS database included 57 patients who were compound heterozygotes carrying known non‐GAA *FXN* mutations (Table 1), including 8 mutations not previously reported and 8 variants that were categorized as partial function. Patients examined at sites in Europe for the European Friedreich's Ataxia Consortium for Translational Studies (EFACTS) were not part of this dataset. The cohort examined in more detail for this study included 409 patients, comprised of 23 (5.6%) patients with minimal/no function mutations, 28 (6.8%) with partial function mutations, and 358 (87.5%) homozygous individuals. While sex and ambulatory status were similar across the cohort, the groups differed markedly in most baseline characteristics and features (Table 2). On average, individuals with partial function mutations were older, had a later age of onset, and less severe disease whereas individuals with minimal/no function mutations were younger with earlier‐onset, more severe disease. Both non‐GAA groups had higher rates of diabetes (26% and 25% for minimal/no function and partial function, respectively) and optic atrophy (30% and 18%) compared to those of the homozygous patients (8% for both features). However, on multivariate analysis accounting for age and GAA repeat number, only minimal/no function individuals had a significantly increased likelihood of optic atrophy (OR: 1.53, *P* = 0.016; Table S1). The minimal/no function group also had slightly higher rates of diabetes, but point mutation status was not a significant independent predictor of diabetes (OR: 1.08, *P* = 0.098 for minimal/no function; OR: 0.69, *P* = 0.210 for partial function).

**Table 1:** Compound heterozygote *FXN* mutations in FA‐COMS.

Mutation	Number of individuals	Functional classification	Mutation classification rationale
G130V	18	Partial	In vitro and modeling data
c.11_12delTC	4	Minimal/none	Early mutation; predicted truncation
L106S	3	Minimal/none	Significant destabilizing effects in models
I154F	3	Partial	In vitro and modeling data
165+5G>C	3	Partial	Splice site; unknown in vitro features
R165C	3	Partial	In vitro and modeling data; inactivating
del. exon 5	3	Minimal/none	Large deletion with truncation
c.2delT	2	Minimal/none	Start codon; predicted incorrect initiation
c.214_215del^1^	2	Minimal/none	Predicted frameshift
c.1A>C	1	Minimal/none	Start codon; predicted incorrect initiation
c.100delG	1	Minimal/none	Early mutation; predicted truncation
c.166‐2A>G^1^	1	Partial	Splice site; unknown features
del. exon 2, 3	1	Minimal/none	Large deletion with truncation
Y143H^1^	1	Partial	Unknown in vitro features
L156P	1	Minimal/none	Significant destabilizing effects in models
W155R	1	Partial	In vitro data; inactivating effects
c.482 del. A+3ins4^1^	1	Partial	Splice site; unknown in vitro features
W168R	1	Minimal/none	In vitro data; severe processing defect
L182F	1	Minimal/none	Significant destabilizing effects in models
285T>A^1^	1	Minimal/none	Stop codon; predicted incorrect termination
del. exon unknown	1	Not classified	
c.371_376del6ins15	1	Not classified	
c.385‐1G>C^1^	1	Not classified	
L140P^1^	1	Not classified	
c.483‐1G>T^1^	1	Not classified	

^1^ Not previously reported in literature.

**Table 2:** Baseline cohort characteristics and clinical features.

	Minimal/no function mutations heterozygous (*n* = 23)	Partial function mutations heterozygous (*n* = 28)	Homozygous (*n* = 358)	*P* value
Age in years, mean (SD)	21.2 (10.9)	33.4 (15.3)	28.2 (13.6)	**0.004**
Female, *n*	8 (35%)	13 (46%)	187 (52%)	0.238
Shorter GAA repeat length, mean (SD)	805 (187)	768 (218)	668 (229)	**0.005**
Age of FRDA onset in years, mean (SD)	8.0 (8.4)	13.0 (6.4)	11.9 (7.7)	**0.039**
Disease duration in years, mean (SD)	13.5 (7.2)	21.2 (15.9)	16.3 (9.7)	**0.018**
mFARS, mean (SD)	59.2 (18.8)	43.2 (15.1)	51.9 (18.8)	**0.009**
Ambulatory, *n*	11 (48%)	16 (57%)	202 (56%)	0.717
Diabetes, *n*	6 (26%)	7 (25%)	29 (8%)	**0.001**
Optic atrophy, *n*	7 (30%)	5 (18%)	28 (8%)	**0.001**
RNFL thickness, mean (SD)	66.7 (16.8)	83.3 (17.0)	73.7 (13.4)	**0.001**
Scoliosis, *n*	16 (70%)	18 (64%)	295 (82%)	**0.027**
Fusion surgery, *n*	9 (39%)	2 (7%)	89 (25%)	**0.027**
Hearing loss, *n*	6 (26%)	4 (14%)	138 (39%)	**0.021**
Frataxin (blood), mean (SD)	44.9 (45.2)	12.5 (9.9)	21.1 (14.1)	**<0.001**
Frataxin (buccal), mean (SD)	12.9 (6.9)	13.4 (10.0)	25.4 (20.2)	**0.003**
Omaveloxolone trial participant, *n*	1 (4%)	2 (8%)	32 (9%)	

Bolded values indicate significance at *P* < 0.05.

Analysis of specific mutation types reveals further underlying patterns (Table S2). Among minimal/no function mutations, five individuals with destabilizing variants (L106S, L165P) had particularly early‐onset disease with a range of 3–6 years. Of the seven individuals with mutations in the start codon or early in the *FXN* gene (c.1A>C, c.2delT, c11_12delTC, and c100delG), three had diabetes while only two had scoliosis, and frataxin levels in the blood were notably much higher (average of 88 compared to a range of 10–20 for all other subgroups). Other point mutation subgroups—both minimal/no function and partial function—were associated with lower average frataxin levels in both the blood and buccal cells compared to homozygous.

For partial function mutations, G130V individuals were far more likely to be ambulatory than their counterparts (83% vs. 10%), and they had consistently less severe disease overall (Table S3). Other types of partial function mutations (I154F, R165C, splice site donor and recipient site mutations, and Y143H) represented the vast majority of cardiac disease, diabetes, and optic atrophy among the entire partial function group.

The atypical FRDA phenotype in certain compound heterozygote individuals is most apparent when examining certain disease characteristics (Table 3). All partial function mutations were associated with a lack of speech or bulbar involvement, with the exception of two individuals with splice site mutations who had minimal dysarthria. Partial function mutations were also associated with preserved upper limb coordination and deep tendon reflexes, with almost universal sparing of these features in G130V individuals. Paradoxically, three of four patients with R165C or 165+5 G>C mutations had unusually rapid‐progressing and severe optic atrophy despite relatively mild disease features otherwise. Three of these four patients also had diabetes, two of whom represented the only early‐onset cases in the partial function group overall.

**Table 3:** Distinctive clinical features of compound heterozygotes.

	Null mutations heterozygous (*n* = 23)	Missense mutations heterozygous (*n* = 28)	Homozygous (*n* = 358)
Dysarthria/bulbar dysfunction Moderate to severe: FARS.A > 1	9 (39%) mild 7 (30%) moderate to severe	2 splice site mutations with mild dysarthria All others (93%) unaffected speech, including 4 with disease duration >40 years	113 (32%) mild 37 (10%) moderate to severe
Upper limb coordination	All affected, 18 with at least moderate involvement	16 (57%) unaffected All G130V either with none or minimal involvement	6 (1.7%) unaffected
Deep tendon reflexes Late disease onset: age >24 years	Hyperreflexia present in 2 atypical *FXN* mutations (c.100delG, c.2delT) Most areflexic in all limbs (22/25)	Most G130V with lower limb reflexes (14/18, most hyperreflexic) and intact in upper limb (15/18) All other mutations with absent lower limb reflexes (0/10) and occasional upper limb (3/10)	Occasional reflexes in late disease‐onset group (6/20 upper, 8/20 lower) Rare (<4%) in all other groups
Optic atrophy Inactive *FXN*: R165 C and 165 + 5 G > C	7 (30%) diagnosed	Severe, with rapid progression in inactive *FXN* mutations (3/4) Rare (1/18) in G130V individuals	28 (7.8%) diagnosed
Diabetes	All 6 with early‐onset at mean age of 11.5 (range: 8–16)	2 early‐onset, both inactive *FXN* mutations 5 late‐onset	
Cardiac disease	3 deceased at age <30 due to cardiac causes	All G130V (18/18) with no clinical or imaging evidence of hypertrophy	

Clinical features separated by mutation groups.

In contrast, the minimal/no function mutation group exhibited a more severe phenotype in nearly all of these domains compared to the homozygous group, with higher rates of both mild and more severe bulbar dysfunction (69% vs. 42% total), optic atrophy, as well as early onset of diabetes with ages ranging from 8 to 16. On multivariate analysis again accounting for age and GAA repeat number, minimal/no function mutation status independently predicted dysarthria (OR: 1.5, *P* = 0.009; Table S1) whereas partial function mutation status had the opposite effect (OR: −4.09, *P* < 0.001).

### Neurological and visual disease outcomes in compound heterozygotes

**Figure 1:**
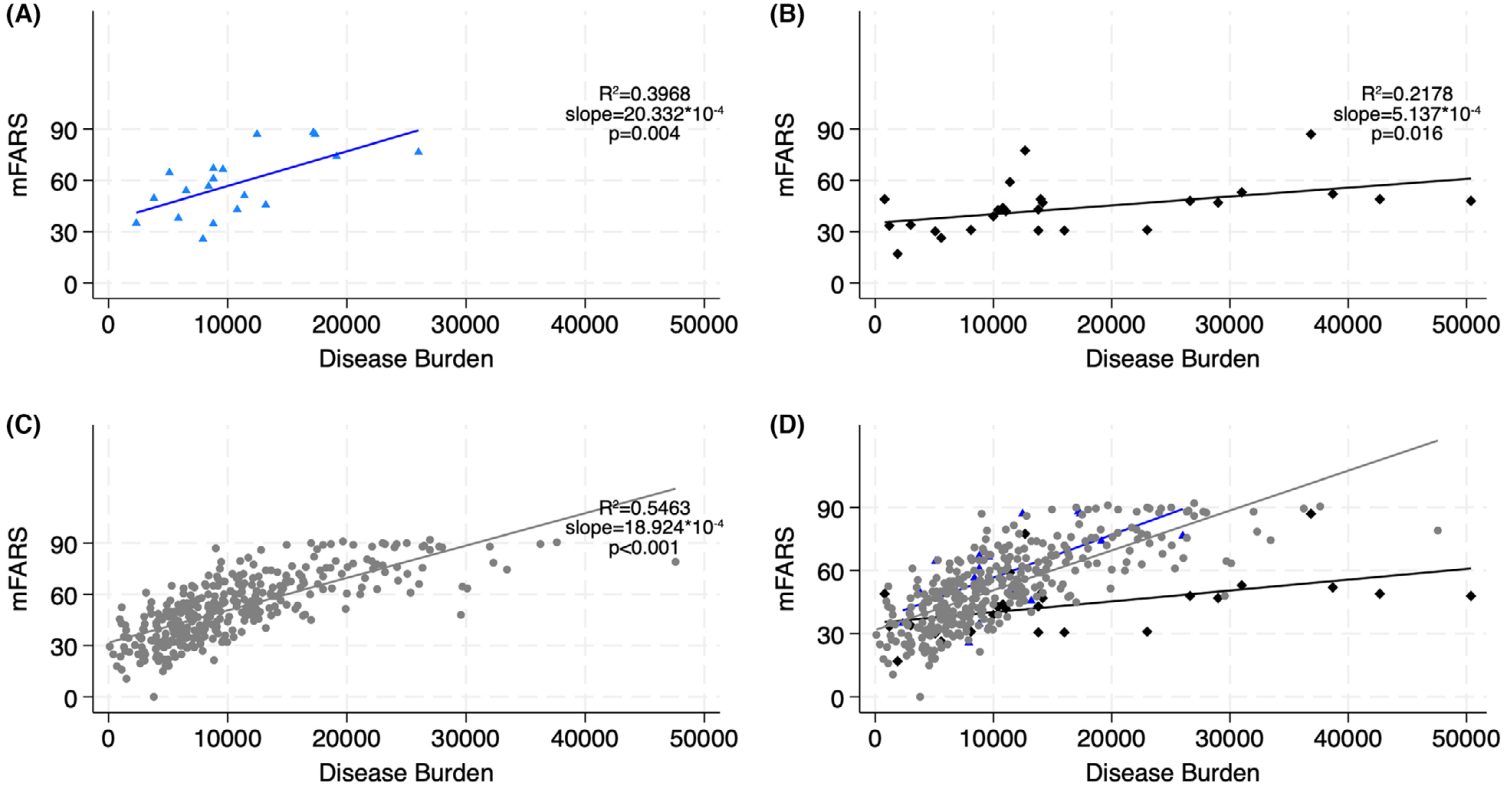
Neurological disease severity among compound heterozygous versus homozygous FRDA patients. mFARS scores from the most recent annual clinical visit plotted against disease burden [GAA length multiplied by disease duration] for (A) minimal/no function mutation heterozygotes, (B) partial function mutation heterozygotes, (C) homozygotes, and (D) overlay of all groups.

**Figure 2:**
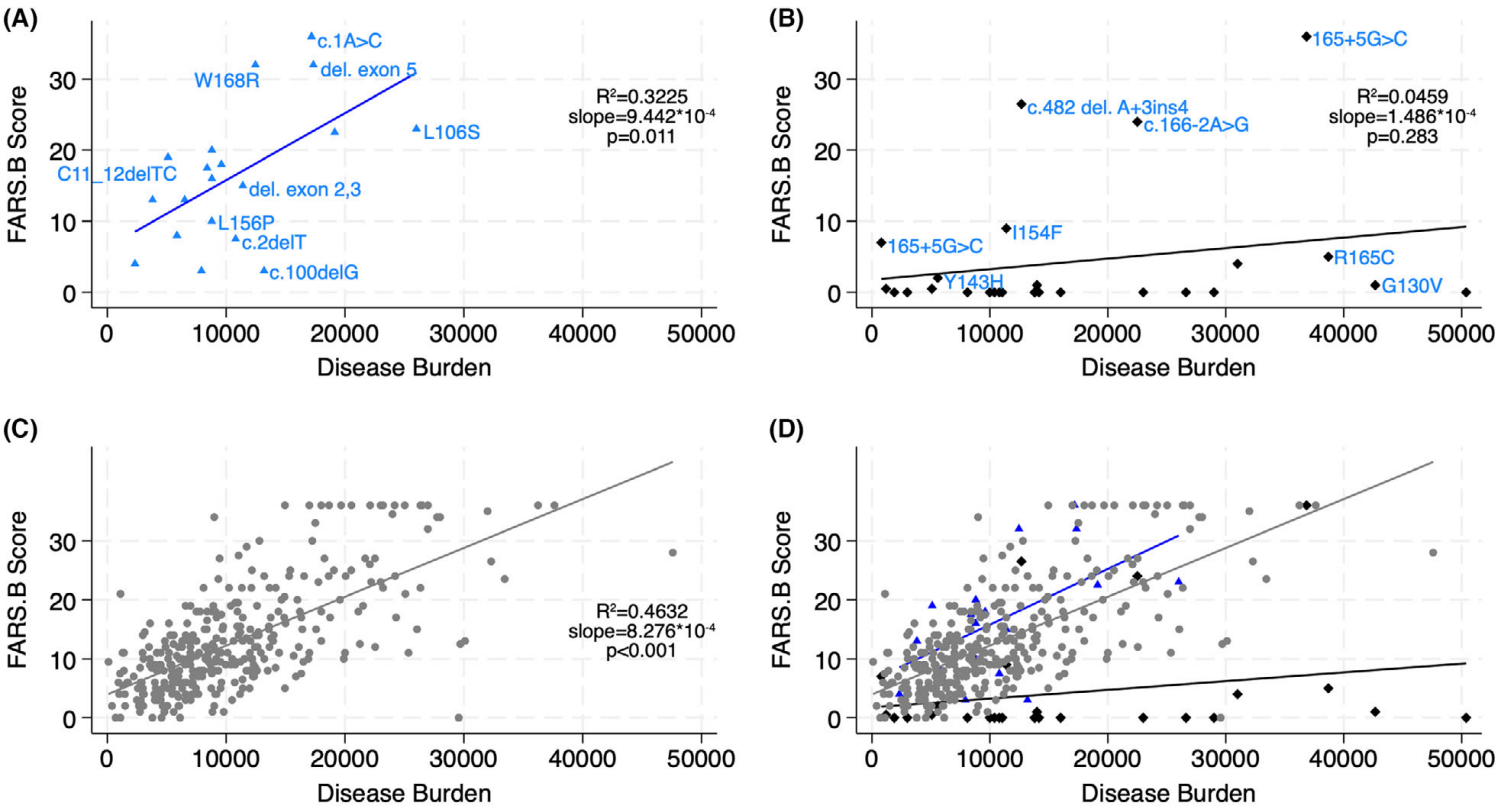
Upper limb coordination is relatively preserved in partial function mutation individuals. Subpart B subscores of the FARS exam shown against disease burden [GAA length multiplied by disease duration] for (A) minimal/no function mutation heterozygotes, (B) partial function mutation heterozygotes, (C) homozygotes, and (D) overlay of all groups. Selected mutations corresponding to individuals are labeled in A and B.

The patterns of total mFARS scores and the FARS B subscores (upper limb coordination) show slightly more severe disease in the minimal/no function mutation than the homozygous group when compared at similar disease burdens (Figs. 1A–D and 2A–D). On the other hand, partial function mutation individuals demonstrate lower mFARS and far lower FARS B subscores than expected based on their disease burden; the majority had minimal to no upper limb involvement with the exception of three patients with splice site mutations (but one of whom was observed over a long disease duration of 55 years).

Cross‐sectional FARS C (lower limb coordination) scores were similar across all groups in the ambulatory subsets, which were used since nonambulatory individuals have near‐maximum or maximum scores (Figure S1A–D). Cross‐sectional FARS E (upright stability) scores among ambulatory patients were also comparable although the homozygous group had modestly higher scores when accounting for disease burden (Figure S2A–D); this difference is likely driven by a larger number of borderline ambulatory individuals (whereas those with minimal/no function mutations rapidly progress to becoming nonambulatory). Taken together, these findings suggest that the mFARS differences are primarily driven by upper limb progression, with FARS A subscores (bulbar function) playing a more minor role as A contributes only a small fraction to the overall mFARS score.

**Figure 3:**
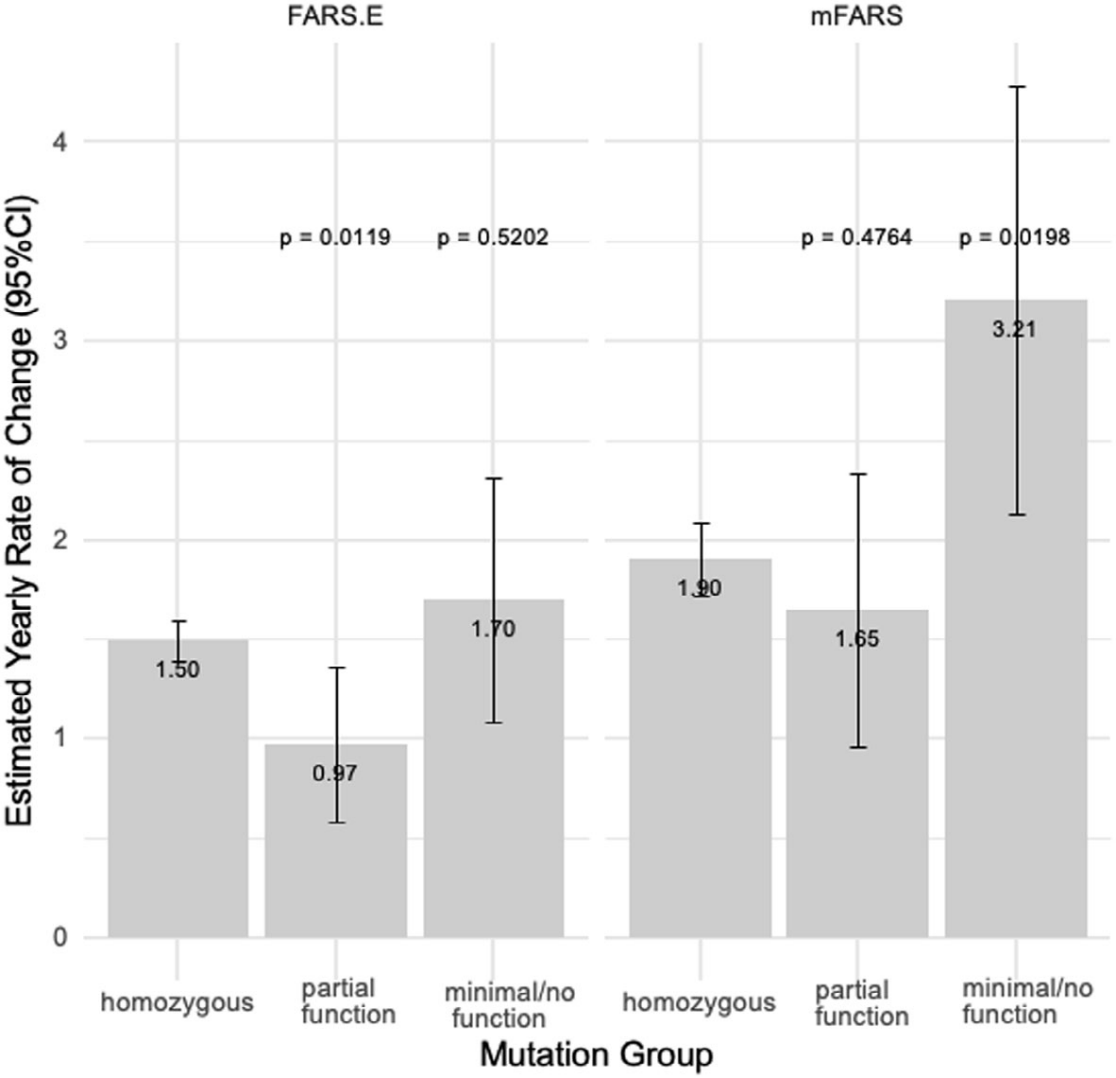
Predicted annual rate of neurological progression by mutation group. Estimated changes in score per year from subpart E of the mFARS are displayed on the left and the total scores on the right. *P*‐values are given for the difference in progression compared to the reference group (homozygous).

Analyses of long‐term neurological progression in mFARS and FARS E indicated other differences (Fig. 3). In FARS E, the partial function group progressed significantly slower than the homozygous group (0.97 vs. 1.50 points per year), and in mFARS the minimal/no function mutation group had significantly faster progression than the homozygous group (3.21 vs. 1.90 points per year). Other differences were clinically relevant, although, likely due to the small group sizes, statistical significance was not always shown (Table S5).

**Figure 4:**
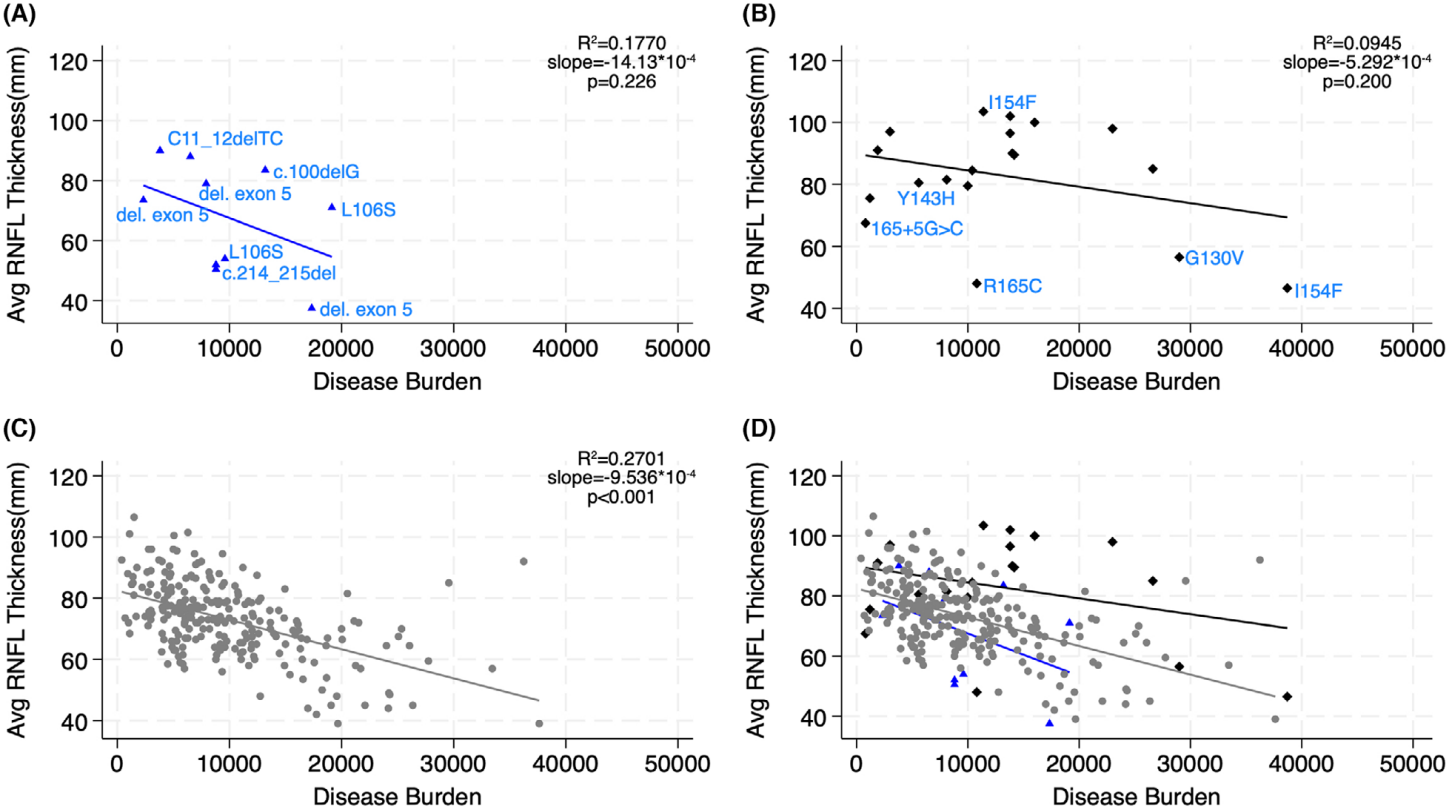
RNFL thickness in compound heterozygous versus homozygous FRDA patients. The average of both eyes' RNFL as measured by OCT plotted against disease burden [GAA length multiplied by disease duration] in (A) minimal/no function mutation heterozygotes, (B) partial function mutation heterozygotes, (C) homozygotes, and (D) overlay of all groups. Selected mutations corresponding to individuals are labeled in A and B.

A comparison of average retinal nerve fiber layer thickness measured by OCT mirrors the pattern predicted by optic atrophy prevalence across the three groups (Fig. 4A–D). Individuals with minimal/no function mutations had worse RNFL measurements than expected based on disease burden while those with partial function mutations had relatively better ones. There was not a clear predilection for a specific subgroup of minimal/no function mutation.

### Detailed cardiac measures in compound heterozygotes

Partial function mutation individuals had less cardiac involvement across multiple measures whereas minimal/no function mutations were associated with variable presentations (Table 4). On multivariate analysis, the presence of either type of mutation was significantly associated with a lower likelihood of cardiac disease (OR: −2.98, *P* < 0.001 for partial function; OR: −0.99, *P* < 0.046 for minimal/no function; Table S1) although this difference was far greater for partial function variants.

**Table 4:** Cardiac disease measures in compound heterozygotes.

	Minimal/no function mutations heterozygous	Partial function mutations heterozygous	Homozygous	*P* value
Cardiac disease, *n*	11 (48%)	3 (11%)	233 (65%)	**<0.001**
Elevated troponin, *n*	5 (36%)	1 (7%)	114 (43%)	**0.020**
Arrhythmia, *n*	3 (13%)	0 (0%)	48 (13%)	0.117
Echo measurements				
Ejection fraction, mean (SD)	58.6 (8.1)	61.7 (4.2)	60.9 (9.0)	0.521
IVS/PWd ratio, mean (SD)	1.08 (0.23)	1.01 (0.16)	1.07 (0.27)	0.707
Relative wall thickness, mean (SD)	0.546 (0.186)	0.359 (0.100)	0.504 (0.407)	0.375
Average E/e′, mean (SD)	8.45 (3.24)	7.18 (1.67)	7.35 (1.97)	0.117
Mitral E/A ratio, mean (SD)	1.91 (0.52)	1.60 (0.52)	1.80 (0.69)	0.565
ECG measurements				
Nonspecific ST/T changes, *n*	11 (69%)	7 (44%)	222 (73%)	**0.037**
PR interval, mean (SD)	153 (77)	133 (17)	137 (20)	**0.041**
QRS interval, mean (SD)	78 (9)	85 (10)	84 (25)	0.622
QT interval, mean (SD)	429 (30)	431 (22)	429 (16)	0.985

Bolded values indicate significance at *P* < 0.05.

However, three individuals with minimal/no function mutations died of cardiac causes before the age of 30. In addition, the minimal/no function mutation group appeared to have consistently poorer average measures across echocardiograms (lower EF, higher RWT, and worse diastolic function measures) although these differences were not statistically significant. Minimal/no function mutation individuals also had comparable rates of arrhythmia and specific ECG abnormalities to those of the homozygous group, with the most common findings on ECG being right axis deviation and right ventricular hypertrophy (Table S4).

Partial function mutation status independently predicted a lower likelihood of elevated troponin (OR: −2.4, *P* = 0.022; Table S1) and lower likelihood of nonspecific ECG changes (OR: −1.36, *P* = 0.014); nearly all ECGs in this group had no specific abnormalities noted either. In other words, individuals with partial function mutations were significantly more likely to have normal troponin levels and normal ECGs.

There were no significant differences in echo measurements across the groups although the pattern was suggestive of more severe disease in the minimal/no function mutation individuals, as previously mentioned, and less severe in the partial function mutation individuals. The groups did differ significantly in the PR interval length, with the minimal/no function group tending to have a longer PR interval (153 vs. 133 for partial function and 137 for homozygous, *P* = 0.041). The QRS and QT intervals were similar. Most Holter monitor readings were close to normal in all groups, with supraventricular ectopy being more common than ventricular ectopy. A minority of homozygous individuals had a history of arrhythmia, compared to two individuals in the minimal/no function group (both L106S) and none in the partial function group (Table S4). The most commonly prescribed cardiac medications were beta‐blockers (80 out of 409, 19.6%), followed by ACE inhibitors or ARBs (47 out of 409, 11.5%) without a clear difference in distribution across the study subgroups.

## Discussion

This study presents detailed analyses of several clinical features in compound heterozygote individuals, including quantitative measures, as compared to typical homozygous FRDA patients. We also identified several novel frataxin mutations, an update from prior summaries.^5^, ^6^, ^7^, ^9^ These findings support more recently posited theories that phenotypical differences in compound heterozygotes are not mediated by their larger average GAA expansions alone. Our methodology, which broadly categorizes *FXN* mutations into either minimal/no function or partial function types, inevitably leads to idiosyncrasies. The partial function category encompasses a diverse array of phenotypic variations, contrasting with the minimal/no function category where predicted effects are more uniform. In turn, these variations exhibit unique characteristics, which are crucial for a deeper understanding of frataxin's role in FRDA pathogenesis.

Specifically, the most compelling evidence in this study suggests that the relationship between frataxin levels versus frataxin dysfunction is not always a simple correlation. Despite having lower average frataxin levels than their homozygous counterparts, partial function mutation individuals had clear sparing of their bulbar and upper limb function on average even at higher disease burdens, a metric combining genetic severity and disease duration.^31^ Deep tendon reflexes and the heart were also relatively preserved (11% with cardiac disease vs. 65% in the homozygous), with complete absence of hypertrophy in 18 G130V individuals even up to disease durations of >50 years. Perhaps most interestingly, the R165C mutation results in close to *normal* frataxin levels,^17^, ^18^, ^27^ yet it and other mutations with similar predicted effects (165+5 G>C, R165P) in both our and prior studies are associated with consistently atypical and especially unusual phenotypes^11^, ^12^; spared features as noted above, but with profound visual loss and diabetes early on in the disease course. Little clinical information exists on patients with the W155R mutation, but it also has inactivating effects on frataxin.^24^, ^25^, ^32^ The known case of homozygosity for R165C is marked by severe visual loss, motor neuropathy, and preserved reflexes thus carrying a different clinical diagnosis altogether of CMT.^13^ One c100delG patient in the present cohort provides another atypical example—the compound heterozygote individual included here had onset of diabetes (Type 1 without islet cell antibodies) more than a decade prior to his neurological diagnosis of FRDA, as well as minimal upper limb involvement and hyperreflexia—although was categorized in the minimal/no function mutation group for the purposes of this study. In the case of G130V patients, our cohort had a relatively low incidence of optic atrophy (1 out of 18) and diabetes (2 out of 18), but these features have been described more often in prior G130V cases.^9^, ^33^ One might argue that our observed incidence is still disproportionately high (i.e., would have expected 0 individuals) in the context of their overall mild disease. It is also possible that the limitations of the present sample size give rise to these results.

These phenotypes raise a question of high translational significance: that there are perhaps specific portions of frataxin that have different functions beyond just those involved in Fe‐S cluster synthesis, with these functions being variably affected by certain mutations. For example, some “partial function” variants may share a preserved part of frataxin that contributes to milder neurologic or cardiac disease but on the other hand, may more severely impair a different frataxin component that is more critical in the visual and/or pancreatic tissues. As a result, current clinical measures of FRDA progression based on homozygous phenotypes may not completely capture their disease. It would appear as though those with partial function mutations have relatively “mild” FRDA based on their FARS exam, as displayed here and in most clinical studies; however, this score does not encapsulate visual symptoms that are among the most functionally limiting for patients, but which are overrepresented among certain compound heterozygotes. Quantitative measures such as RNFL may be useful in this regard.^34^ Optic atrophy was also overrepresented among the minimal/no function mutation group of our cohort after accounting for genetic severity and disease duration. One possible explanation is that most homozygous individuals have subclinical optic atrophy that never progresses to the critical threshold at which it is clinically diagnosed, even later on in life. In contrast, those in the minimal/no function group seem to have disproportionately rapid progression of their neurological disease at an earlier age, which may parallel their visual disease. These findings are still consistent with the idea that both typical homozygous and minimal/no function mutation phenotypes are driven primarily by frataxin deficiency.

Hypertrophic cardiomyopathy is both a hallmark of FRDA and the predominant cause of mortality, as high as >60% in previous reports^35^, ^36^, ^37^, ^38^, ^39^; therefore one of the clinically important observations in our data is that compound heterozygote individuals in this cohort had significantly less cardiac involvement. Interestingly, this trend held even for minimal/no function mutation individuals despite having more severe disease in essentially every other category. As prior reports also support, their disease phenotype mirrors that of the homozygous group but with higher rates and/or faster progression^5^, ^6^, ^7^, ^8^—including optic atrophy, dysarthria, neurological disease including upper limb coordination, severe scoliosis, and diabetes (albeit without a significant difference in diabetes among our cohort, unlike the previous study).^5^ The exception is a paradoxically lower rate of cardiac disease (48% vs. 65% in the homozygous). Upon further examination of detailed cardiac measures though, minimal/no function individuals have slightly but consistently poorer metrics on echocardiogram although the ranges are difficult to interpret statistically. Moreover, a striking number of individuals (3 out of 23, 13%) in this group died before the age of 30 of cardiac causes. It is possible that those who do exhibit cardiac involvement with minimal/no function mutations tend to have particularly severe disease, suggesting that the overall pattern should be interpreted with caution. For instance, there may have been other unknown individuals with early cardiac mortality, who might have otherwise been followed at a study site later on, potentially biasing the sample studied. Case reports suggest that the pathological phenotypes in cardiac tissue of two patients with a c11_12delTC and exon 5 deletion, is largely indistinguishable from those of age‐matched homozygous counterparts and is marked by similar cardiomyocyte hypertrophy and iron‐positive inclusions.^40^

Relative sparing of the heart in partial function mutations has been observed in previous studies, which our data support.^5^, ^9^ In addition, this group was also significantly more likely to have normal troponin levels and normal ECG findings, suggesting that these measures (as shown here in compound heterozygotes) may have a meaningful correlation with clinical disease. Still, nonspecific or ischemic‐like T‐wave changes on ECG were not uncommon (7 out of 16, 44%) in this group. As has been previously suggested though, such changes are unlikely to be clinically useful (as they do not change substantially over time)^41^ and do not contradict other reassuring findings, like normal troponin levels and the absence of other specific ECG abnormalities, that might warrant less aggressive cardiac follow‐up in these patients. Chronically elevated troponins have been recognized in FRDA patients, but their clinical meaning is unclear.^42^, ^43^

Individuals with partial function variants did exhibit a trend of echocardiogram measures indicating more normal values compared to homozygous patients, but echo data remain challenging to correlate clinically.^44^, ^45^, ^46^, ^47^ Previously, only LVEF has been a reliable predictor for poor long‐term outcomes, and LVEF tends to decline markedly only in very advanced disease.^35^, ^48^ As for ECG data, the slightly longer PR interval in minimal/no function mutation individuals could reflect early stages of fibrosis, usually a later feature of cardiomyopathy in FRDA.^37^, ^38^ While this difference is not likely to be directly significant, the natural history of cardiac disease in FA remains relatively understudied, and research is ongoing to improve markers for predicting progression.^45^, ^46^, ^47^ Arrhythmia and Holter data were limited but do not appear to vary dramatically among compound heterozygotes compared to their homozygous counterparts here and in prior data,^49^ apart from a higher incidence of arrhythmia in this cohort's L106S patients (2 out of 4, 50%; the only instances of arrhythmia in the minimal/no function mutation group).

There are limitations of the study resulting from selective availability of clinical data. Although most patients are recommended to receive formal cardiac evaluation annually, many either did not do so, or did not have updated studies. The cardiac data are probably skewed toward appearing more severe as patients who never experienced cardiac symptoms and/or have never had identifiable hypertrophy would be less likely to follow‐up. The prevalence of compound heterozygotes was also quite high in our cohort (~10%) compared to general population estimates at closer to 4%,^3^ even after excluding the additional individuals added outside the main cohort study window. This disparity likely reflects the types of patients seen at a large, specialized tertiary referral center, and there may have been other characteristics associated with this group that would limit broader generalizability.

Several questions remain surrounding the exact functions of frataxin. Larger studies might help illuminate whether clinical features are consistent across individuals with frataxin mutations. This information may reveal additional insight into the characteristics of specific mutation types, which was limited in our study due to small sample sizes; for example, L106S was categorized as a minimal/no function mutation here due to its destabilizing effects,^5^, ^22^ but it is unclear whether the phenotype might have any reliably distinguishing features (our preliminary data are suggestive of very early‐onset disease and possibly more arrhythmia). We grouped mutations here to increase samples for primary analyses, but these classifications have limitations as some mutations likely do not fit neatly into a minimal/no function versus partial function binary, such as the cdel100G discussed earlier. Another example is shown by the splice site mutations, with individuals in this study exhibiting more upper limb involvement than those with other mutations classified as “partial function.” Advances in access to genetic sequencing and technology may assist in identification of individuals with atypical‐presenting *FXN* mutations and greater diagnostic clarity, for instance in those who may have otherwise carried a clinical diagnosis of CMT. Future research could further elucidate how impairments in frataxin function or functions leads to disease, with broader applications to restoring that function therapeutically in FRDA patients.

## Author contributions

MMS, CR, and DRL contributed to the conception and design of the study. MMS and CR contributed to acquisition and analysis of data.

## Conflict of interest

The authors report no other conflicts of interest.

## Supporting information


**Figure S1.** Lower limb coordination is similar among ambulatory compound heterozygotes and homozygotes. Subpart C subscores of the FARS exam plotted against disease burden [GAA length multiplied by disease duration] for (A) minimal/no function mutation heterozygotes, (B) partial function mutation heterozygotes, (C) homozygotes, and (D) overlay of all groups.
**Figure S2.** Upright stability is comparable among ambulatory compound heterozygotes and homozygotes. Subpart E subscores of the FARS exam plotted against disease burden [GAA length multiplied by disease duration] for (A) minimal/no function mutation heterozygotes, (B) partial function mutation heterozygotes, (C) homozygotes, and (D) overlay of all groups.
**Table S1.** Multivariate analysis by mutation group compared to reference (homozygous group), accounting for GAA repeat number and age. Confidence intervals are reported at 95%.
**Table S2.** Characteristics of minimal/no function mutation patients by subtype. The deletions group was comprised of large deletions involving an exon while the start codon/early mutation group included c.1A>C, c.2delT, c11_12delTC, and c100delG, and the destabilizing group included L106S and L156P.
**Table S3.** Characteristics of partial function mutation patients by subtype. Variants included are listed in Table 1.
**Table S4.** Detailed descriptive cardiac data by mutation group including specific types of dysfunction, findings on EKG and Holter monitors, and cardiac medication types.
**Table S5.** Predicted annual rate of neurological progression by mutation group as measured by the mFARS and FARS E/upright stability scores.
