# Supplementary material for: Phenotypic variation of FXN compound heterozygotes in a Friedreich ataxia cohort
Source: Ann Clin Transl Neurol. 2024 Feb 23;11(5):1110–21. doi: 10.1002/acn3.52027 (PMC11093247; doi:10.1002/acn3.52027)

|  | Minimal/no function | Partial function |
| --- | --- | --- |
| Cardiac disease | **-0.99 (-1.95, -0.02)**  **p=0.046** | **-2.98 (-4.24, -1.72)**  **p<0.001** |
| Diabetes mellitus | 1.08 (-0.20, 2.35)  p=0.098 | 0.69 (-0.39, 1.75)  p=0.210 |
| Optic atrophy | **1.53 (0.29, 2.78)**  **p=0.016** | -0.12 (-1.37, 1.13)  p=0.853 |
| Speech involvement/dysarthria | **1.50 (0.28, 2.62)**  **p=0.009** | **-4.09 (-5.87, -2.30)**  **p<0.001** |
| Elevated troponin | -0.59 (-1.85, 0.66)  p=0.356 | **-2.40 (-4.45, -0.35)**  **p=0.022** |
| ECG T-wave nonspecific changes | -0.26 (-1.47, 0.95)  p=0.675 | **-1.36 (-2.45, -0.27)**  **p=0.014** |

**Supplementary Table 1.** Multivariate analysis by mutation group compared to reference (homozygous group), accounting for GAA repeat number and age, with confidence intervals reported at 95%.

**Supplementary Table 2.** Characteristics of minimal/no function mutation patients by subtype.

|  | Exon deletions  (n=6) | Start codon/early mutations  (n=7) | Destabilizing Mutations (n=5) |
| --- | --- | --- | --- |
| Age in years, mean (SD) | 20.2 (5.4) | 25.6 (16.7) | 21.4 (9.4) |
| Female, n | 4 (67%) | 1 (14%) | 2 (40%) |
| Shorter GAA repeat length, mean^b^ (SD) | 625 (171) | 785 (112) | 806 (126) |
| Age of FRDA onset in years, mean (SD)  Range | 7.8 (3.9)  3-13 | 13.1 (13.9)    4-41 | 4.6 (1.1)  3-6 |
| mFARS, mean (SD) | 50.4 (22.9) | 59.0 (15.8) | 61.6 (17.0) |
| Ambulatory, n | 3 (50%) | 4 (57%) | 2 (40%) |
| Cardiac disease | 3 (50%) | 4 (57%) | 2 (40%) |
| Diabetes, n | 0 (0%) | 3 (43%) | 1 (20%) |
| Optic Atrophy, n | 1 (17%) | 1 (14%) | 2 (40%) |
| RNFL thickness,  mean (SD) | 63.9 (18.4) | 79.4 (15.8) | 62.5 (12.0) |
| Scoliosis, n | 4 (67%) | 2 (29%) | 5 (100%) |
| Fusion surgery, n | 3 (50%) | 1 (14%) | 3 (60%) |
| Hearing Loss, n | 2 (33%) | 1 (14%) | 2 (40%) |
| Frataxin (blood),  mean (SD) | 19.8 (0.6) | 88.0 (33.5) | 10.7^a^ |
| Frataxin (buccal),  mean (SD) | 10.5 (4.6) | 12.8 (5.8) | 13.6 (12.5) |
| Omaveloxolone trial participant, n | - | 1 | - |

^a^n=1 sample available

|  | G130V  (n=18) | Other partial function variants  (n=10) |
| --- | --- | --- |
| Age in years, mean (SD) | 33.0 (15.3) | 36.2 (15.7) |
| Female, n | 9 (50%) | 4 (40%) |
| Shorter GAA repeat length, mean^b^ (SD) | 835 (141) | 655 (283) |
| Age of FRDA onset in years, mean (SD)  Range | 14.3 (6.5)  6-30 | 10.8 (5.7)  5-21 |
| mFARS, mean (SD) | 37.8 (9.5) | 54.1 (18.7) |
| Ambulatory, n | 15 (83%) | 1 (10%) |
| Cardiac disease | 0 (0%) | 3 (30%) |
| Diabetes, n | 2 (11%) | 5 (50%) |
| Optic Atrophy, n | 1 (6%) | 4 (40%) |
| RNFL thickness,  mean (SD) | 88.6 (11.9) | 70.9 (21.7) |
| Scoliosis, n | 11 (61%) | 7 (70%) |
| Fusion surgery, n | 0 (0%) | 2 (20%) |
| Hearing Loss, n | 2 (11%) | 2 (20%) |
| Frataxin (blood),  mean (SD) | 12.2 (10.4) | 13.0 (9.8) |
| Frataxin (buccal),  mean (SD) | 10.3 (6.7) | 18.5 (13.0) |
| Omaveloxolone trial participant, n | 1 | 1 |

**Supplementary Table 3.** Characteristics of partial function mutation patients by subtype.

**Supplementary Table 4**. Detailed descriptive cardiac data.

|  | Minimal/no function mutations Heterozygous | Partial function mutations  Heterozygous | Homozygous |
| --- | --- | --- | --- |
| Dilated cardiomyopathy^a^ | - | - | 4 |
| Systolic dysfunction^a^  EF<50% | 2 | - | 22 |
| Arrhythmia | 2, both L106S | - | 50 |
| Ablation | - | - | 8 |
| ICD | - | - | 3 |
| Pacemaker | 1 | - | 1 |
|  | | | |
| EKG findings^b^ |  |  |  |
| R axis | 2 (13%) | - | 65 (21%) |
| R ventricular hypertrophy | 1 (6.3%) | - | 27 (8.9%) |
| R atrial enlargement | 2 (13%) | - | 5 (1.7%) |
| L atrial enlargement | 1 (6.3%) | 1 (6.3%) | 16 (5.3%) |
| L ventricular hypertrophy | 2 (13%) | - | 16 (5.3%) |
| L axis | - | - | 11 (3.6%) |
| Conduction abnormality  (ie bundle branch block) | 1 (6.3%) | - | 20 (6.6%) |
| Biatrial enlargement | 1 (6.3%) | - | - |
| Biventricular hypertrophy | 1 (6.3%) | - | 6 (2.0%) |
| ST elevations | - | - | 12 (4.0%) |
| Low voltage | - | - | 12 (4.0%) |
|  | | | |
| Holter findings^c^ |  |  |  |
| Average rate (SD) | 89 (9.1) | 91(8.6) | 85(11) |
| Supraventricular ectopy (n) | 6 (55%) | 2 (50%) | 132 (83%) |
| Rare | 5 | 2 | 113 |
| Occasional | - | - | 9 |
| Frequent | 1 | - | 10 |
| NSVT runs present (n) | - | - | 31 |
| Ventricular ectopy (n) | 3 (27%) | 3 (75%) | 83 (52%) |
| Rare | 2 | 3 | 76 |
| Occasional | 1 | - | 6 |
| Frequent | - | - | 1 |
|  | | | |
| Cardiac Medications (n) |  |  |  |
| Beta-blocker | 3 | 1 | 77 |
| Ace inhibitor/ARB | 2 | 2 | 43 |
| Entresto | - | - | 8 |
| Calcium-channel blocker | - | - | 11 |
| Other anti-arrhythmic | - | - | 9 |
| MCR antagonist | - | - | 14 |
| SGLT2 inhibitor | - | - | 4 |
| Loop diuretic | - | - | 7 |

^a^EFs available for n=17, n=13, n=288

^b^EKG data available for n=16, n=16, n=303

^c^Holter data available for n=11, n=4, n=160

**Supplementary Table 5.** Predicted annual rate of neurological progression by mutation group.

| Scale | Group | Rate of Progression (95%CI) | p-value | Group Difference | p-value |
| --- | --- | --- | --- | --- | --- |
| FARS E | homozygous | 1.50 (1.39, 1.60) | <0.0001 |  |  |
| FARS E | partial function | 0.97 (0.58, 1.36) | <0.0001 | -0.52 (-0.93, -0.12) | 0.0119 |
| FARS E | minimal/no function | 1.70 (1.09, 2.31) | <0.0001 | 0.20 (-0.42, 0.82) | 0.5202 |
| mFARS | homozygous | 1.90 (1.72, 2.09) | <0.0001 |  |  |
| mFARS | partial function | 1.65 (0.96, 2.33) | <0.0001 | -0.26 (-0.97, 0.45) | 0.4764 |
| mFARS | minimal/no function | 3.21 (2.13, 4.28) | <0.0001 | 1.30 (0.21, 2.39) | 0.0198 |

**Supplementary Figure 1.** Lower limb coordination is similar among ambulatory compound heterozygotes and homozygotes. Subpart C subscores of the FARS exam plotted against disease burden [GAA length multiplied by disease duration] for (A) minimal/no function mutation heterozygotes, (B) partial function mutation heterozygotes, (C) homozygotes, and (D) overlay of all groups.


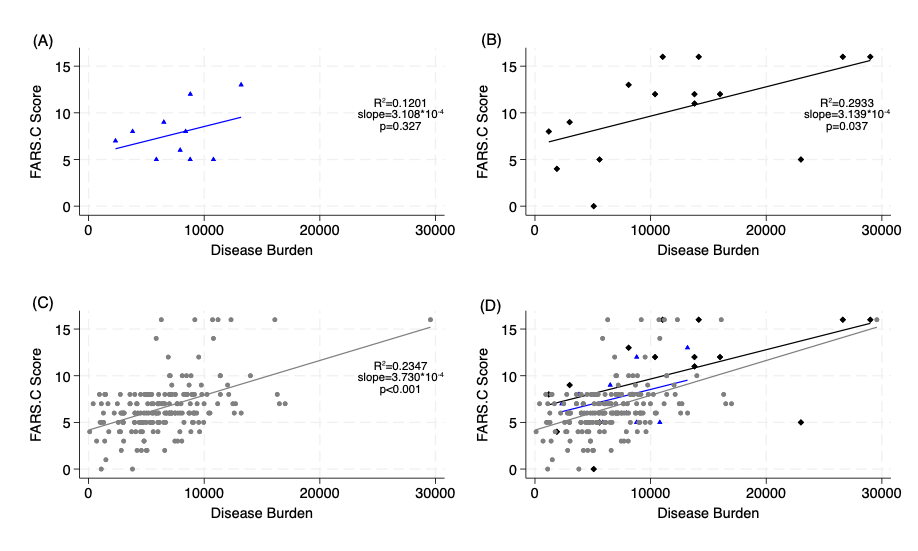


**Supplementary Figure 2.** Upright stability is comparable among ambulatory compound heterozygotes and homozygotes. Subpart E subscores of the FARS exam plotted against disease burden [GAA length multiplied by disease duration] for (A) minimal/no function mutation heterozygotes, (B) partial function mutation heterozygotes, (C) homozygotes, and (D) overlay of all groups.


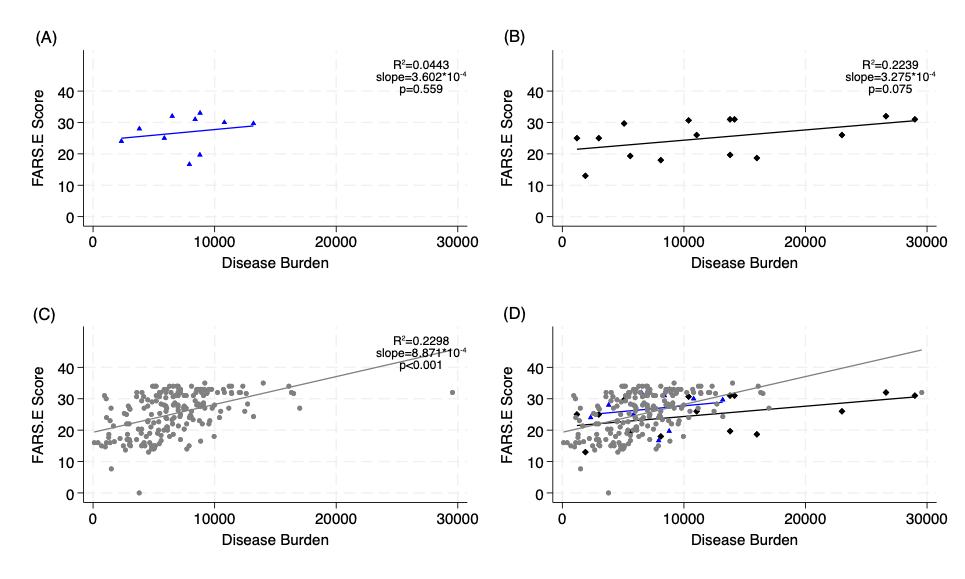

Supplement: Supplementary file 1 — Figure S1. Lower limb coordination is similar among ambulatory compound heterozygotes and homozygotes. Subpart C subscores of the FARS exam plotted against disease burden [GAA length multiplied by disease duration] for (A) minimal/no function mutation heterozygotes, (B) partial function mutation heterozygotes, (C) homozygotes, and (D) overlay of all groups. Figure S2. Upright stability is comparable among ambulatory compound heterozygotes and homozygotes. Subpart E subscores of the FARS exam plotted against disease burden [GAA length multiplied by disease duration] for (A) minimal/no function mutation heterozygotes, (B) partial function mutation heterozygotes, (C) homozygotes, and (D) overlay of all groups. Table S1. Multivariate analysis by mutation group compared to reference (homozygous group), accounting for GAA repeat number and age. Confidence intervals are reported at 95%. Table S2. Characteristics of minimal/no function mutation patients by subtype. The deletions group was comprised of large deletions involving an exon while the start codon/early mutation group included c.1A>C, c.2delT, c11_12delTC, and c100delG, and the destabilizing group included L106S and L156P. Table S3. Characteristics of partial function mutation patients by subtype. Variants included are listed in Table 1. Table S4. Detailed descriptive cardiac data by mutation group including specific types of dysfunction, findings on EKG and Holter monitors, and cardiac medication types. Table S5. Predicted annual rate of neurological progression by mutation group as measured by the mFARS and FARS E/upright stability scores. [file ACN3-11-1110-s001.docx]
